# Supplementary material for: The transcription factors Runx3 and ThPOK cross-regulate acquisition of cytotoxic function by human Th1 lymphocytes
Source: eLife. 2018 Feb 28;7:e30496. doi: 10.7554/eLife.30496 (PMC5844691; doi:10.7554/eLife.30496)
Supplement: Supplementary file 2. — The level of methylation was measured by pyrosequencing in memory Th1 cell subsets of two to nine CMV-seropositive healthy adults. Data are median percentage ± interquartile range and were compared with the Mann-Withney non-parametric test. † CMTh1 versus naive CD4 T cells. ‡ EM28+Th1 versus naive CD4 T cells. § CD4CTX versus naive CD4 T cells. ¶ EM28+Th1 versus CMTh1. || CD4CTX versus EM28+Th1. nd : not done, no statistical analysis was performed because of insufficient number of subjects (n = 2 or 3) ; ns : non significant ;*=p<0.05 ; **=p<0.01 ; ***=p<0.001 ; ****=p<0.0001. (b) Monoclonal antibodies used in flow cytometry experiments. (c). QPCR Oligonucleotide sequences (d) ChIP-PCR oligonucleotide sequences. (e) Single-cell qPCR oligonucleotide sequences. (f) References for the gene transfer plasmids used in transcription factor knockdown experiments. [file elife-30496-supp2.docx]

**Supplementary File 2a. Level of methylation at individual CpG sites in memory Th1 cell subsets *in vivo***

| **CpG site** | **Naive CD4**  (n = 9) | **CM_Th1_**  (n = 2-6) | **EM28^+^_Th1_**  (n = 5) | **CD4_CTX_**  (n = 8-9) | **p value**  **†** | **p value**  **‡** | **p value**  **§** | **p value**  **¶** | **p value**  **ǁ** |
| --- | --- | --- | --- | --- | --- | --- | --- | --- | --- |
| 1 | 100 (±0.95) | 96.8 (±8.00) | 89.05 (±2.82) | 20.53(±12.21) | ns | ** | **** | * | *** |
| 2 | 95.27 (±2.62) | 92.83 (±6.94) | 91.29 (±1.16) | 33.42(±20.64) | ns | ns | **** | ns | *** |
| 3 | 97.46 (±0.60) | 95.5 (±5.39) | 88.96 (±5.42) | 24.75(±17.35) | ns | *** | **** | ns | *** |
| 4 | 88.35 (±1.14) | 84.3 (±6.00) | 85.22 (±0.75) | 22.32(±11.65) | ns | ** | **** | ns | *** |
| 6 | 96.19 (±0.14) | 91.85 (±9.32) | 87.34 (±3.82) | 13.2 (±12.51) | ns | *** | **** | ns | ** |
| 7 | 94.79 (±0.37) | 81.53 (±6.82) | 72.14 (±9.24) | 2.77 (±1.56) | ** | *** | **** | * | *** |
| 8 | 99.6 (±13.52) | 85.37 (±6.63) | 75.72 (±3.75) | 5.91 (±1.62) | ** | *** | **** | * | *** |
| 9 | 97.6 (±23.97) | 79.34 (±4.56) | 68.1 (±3.15) | 2.97 (±4.00) | ** | *** | **** | * | *** |
| 10 | 94.32 (±0.34) | 72.38 (±8.93) | 58.81 (±6.93) | 6.53 (±3.49) | ** | *** | **** | ns | *** |
| 14 | 95.24 (±1.11) | 43.82 (±29.0) | 62.91 (±9.38) | 5.69 (±3.27) | nd | * | **** | nd | ** |
| 15 | 96.19 (±0.51) | 82.87 (±2.07) | 71.3 (±7.53) | 4.8 (±19.27) | nd | *** | **** | nd | ** |
| 16 | 97.55 (±2.98) | 68.21 (±3.35) | 59.64 (±6.67) | 6.25 (±3.40) | nd | *** | **** | nd | ** |
| 17 | 95.53 (±2.69) | 64.34 (±2.2) | 51.61 (±1.85) | 6.15 (±19.02) | nd | *** | **** | nd | ** |
| 18 | 65.9 (±11.17) | 31.53 (±9.71) | 24.18 (±3.66) | 3.54 (±3.72) | *** | *** | **** | * | ** |
| 19 | 80 (±9.95) | 37.2 (±10.72) | 24.87 (±3.88) | 4 (±1.3) | *** | *** | **** | * | *** |
| 20 | 75.59 (±3.45) | 43.50 (±9.62) | 30.81 (±2.42) | 3.07 (±1.41) | *** | *** | **** | * | ** |
| 21 | 70 (±5.31) | 38 (±10.26) | 25.98 (±5.52) | 3.48 (±1.29) | *** | *** | **** | * | ** |
| 22 | 60.69 (±5.31) | 33.44 (±6.05) | 24.16 (±1.77) | 5.89 (±3.18) | *** | *** | **** | ** | *** |
| 23 | 68.75 (±3.31) | 40.85 (±8.73) | 31.87 (±1.62) | 9.33 (±4.86) | *** | *** | **** | * | *** |
| 24 | 36.34 (±3.67) | 19.45 (±1.11) | 12.42 (±2.96) | 3.52 (±0.79) | *** | *** | **** | ** | *** |
| 25 | 4.51 (±0.67) | 4.95 (±7.54) | 8.12 (±2.85) | 2.64 (±1.03) | ns | ** | ** | ns | *** |
| 26 | 20.81 (±5.89) | 17.25 (±8.58) | 12.58 (±3.51) | 1.83 (±1.57) | ns | ** | **** | ns | *** |
| 27 | 24.16 (±8.68) | 19.12 (±0.64) | 11.1 (±2.52) | 2.68 (±1.02) | * | *** | **** | ** | *** |
| 28 | 11.62 (±6.58) | 12.64 (±5.60) | 3.47 (±0.9) | 1.91 (±1.45) | ns | *** | **** | ** | * |
| 29 | 2.16 (±0.78) | 3.94 (±0.36) | 2.88 (±0.81) | 1.81 (±0.2) | ** | ns | * | ns | ** |
| 30 | 2.06 (±0.33) | 2.65 (±0.24) | 2.7 (±0.27) | 1.88 (±0.62) | * | ns | ns | ns | * |
| 31 | 4.44 (±0.91) | 6.75 (±1.17) | 5.72 (±1.66) | 3.46 (±0.36) | ** | ns | ** | ns | ** |
| 32 | 6.08 (±1.79) | 9.47 (±1.56) | 7.29 (±2.76) | 2.41 (±2.03) | *** | ns | ** | ns | ** |
| 33 | 1.05 (±0.15) | 1.58 (±0.23) | 1.22 (±0.63) | 1.05 (±1.18) | * | ns | ns | ns | ns |
| 34 | 5.45 (±1.85) | 6.34 (±1.33) | 4.83 (±1.23) | 4.47 (±1.31) | ns | ns | ns | ns | ns |

The level of methylation was measured by pyrosequencing in memory Th1 cell subsets of 2 to 9 CMV-seropositive healthy adults. Data are median percentage +/- interquartile range and were compared with the Mann-Withney non-parametric test.

† CM_Th1_ versus naive CD4 T cells

‡ EM28^+^_Th1_ versus naive CD4 T cells

§ CD4_CTX_ versus naive CD4 T cells

¶ EM28^+^_Th1_ versus CM_Th1_

ǁ CD4_CTX_ versus EM28^+^_Th1_

nd : not done, no statistical analysis was performed because of insufficient number of subjects (n=2 or 3) ; ns : non significant ;

* = *p* < 0.05 ; ** = *p* < 0.01 ; *** = *p* < 0.001 ; **** = p < 0.0001.

**Supplementary File 2b. Monoclonal antibodies used in flow cytometry experiments**

| **Antibody** | **Fluorochromes** | **Clone** | **Company** |
| --- | --- | --- | --- |
| Active Caspase3 | AF647 | C92-605 | BD |
| CCR4 | Pcy7 | 1G1 | BD |
| CCR5 | FITC, AF647 | HEK/1/85a | Imtec |
| CCR6 | PE, Pcy7, BV605, AF647 | 11A9 | BD |
| CCR7 | PE | 150503 | R&D |
| CD14 | PE | MϕP9 | BD |
| CD16 | PE | NKP15 | BD |
| CD19 | PE | HIB19 | eBiosciences |
| CD25 | ECD | B1.49.9 | Analis |
| CD27 | FITC, PE | M-T271 | BD |
| CD28 | ECD | CD28.2 | Analis |
| CD3 | V500 | UCHT1 | BD |
| CD3ε | FITC | APA1/1 | BD |
| CD4 | V450, V500 | RPA-T4 | BD |
| CD45RO | Pcy7, APCH7 | UCHL1 | BD |
| CD56 | PE | NCAM16.2 | BD |
| CD8 | PE, V500 | RPA-T8 | BD |
| CRTh2 | FITC, AF647 | BM16 | BD |
| CXCR3 | Pcy5, Pcy7, AF700 | 1C6/CXCR3 | BD |
| Eomes | eF660, PE-eF610 | WD1928 | eBiosciences |
| GranzymeB | V450 | GB11 | BD |
| Perforin | FITC, PE, APC | delta G9 | BD or eBiosciences |
| Runx3 | APC | aa186-415 | Lifespan biosciences |
| T-bet | V450, PE, BV786 | O4-46 | BD |
| TCRgd | PE | 11F2 | BD |
| ThPOK | PE | 11H11A14 | Imtec |

**Supplementary File 2c. QPCR Oligonucleotide sequences**

| Gene symbol | 5’-FAM-TAMRA-3’ probe | Forward primer 5’-3’ | Reverse primer 5’-3’ |
| --- | --- | --- | --- |
| *EEF1A1* | AAATAAGCGCCGGCTATGCCCCTG | GCTTCACTGCTCAGGTGAT | GCCGTGTGGCAATCCAAT |
| *EOMES* | TCCACCGCCACCAAACTGAGA | GTGCCCACGTCTACCTGTG | AGCTCAAGAAAGGAAACATGC |
| *PRF1* | CCATGTGGTACACACTCCCCCGC | TGGAGTGCGGCTTCTACAGTT | GCCCTCTTGAAGTCAGGGTG |
| *TBX21* | TGAGGTGAACGACGGAGAGCCA | AACAATGTGACCCAGATGATTG | TGAACTGGGTTTCTTGGAAAGT |
| *ZBTB7B* | CATGCCCGCACTGCCCAGCC | ACCAGGAACGACAAGCTGAA | AGGTCGTAGCTGTGCAGGAA |
| *ZNF683* | TTCCAGCTCTTCCGAGGTGACCA | ATGGCCCTGGGAGGTACA | ACCATGTCTGGAAGTGGTCTG |
| *GZMB* | TGCTACTGCAGCTGGAGAGAAAGGCC | TCCTAAGAACTTCTCCAACGACATC | GCACAGCTCTGGTCCGCT |
| *IFNG* | TCCAACGCAAAGCAATACATGAAC | CTAATTATTCGGTAACTGACTTGA | ACAGTTCAGCCATCACTTGGA |
| *HOPX* | ccacgctgtgcctcatcgcgg | gtcgacaagcacccggatt | ggcgctgcttaaaccatttct |
| *ZEB2* | acaagccagggacagatcagcacca | gctcgagcggcatatggt | acttgcgattacctgctcctt |
| *ASCL2* | CTCGCCCTCCCGCCACGCT | GCCTTACAGAATGTGATCGCG | CTTTCTCACGCCCTCCTTCC |
| *GNLY* | ccctctgagccctctcaccttgtc | cctcaggttgtgtataccttctacag | ggagcctgtgcttcttcc |
| *GZMK* | caaaggccagtctcccactgtgg | gcagcccactgccaatatc | tgagagagagtgtgcgcctaa |
| *CX3CR1* | cgatgatttggctgaggcctg | actagaggccttcaccatgg | agaccacgatgtccccaata |
| *CD4* | acgcaagcccagaggccct | cctcacacagatacgcctgtt | gacctgagcccacagaaatg |
| *TNF* | TGGCCCAGGCAGTCAGATCATC | CCCAGGGACCTCTCTCTAATC | ATGGGCTACAGGCTTGTCACT |
| *CD8A* | cgacacccggaactggctcg | agtgaccgccttgctcct | gttccaggtccgatccag |

**Supplementary File 2d. ChIP-PCR oligonucleotide sequences**

| Name (position) | 5’-FAM-TAMRA-3’ probe | Forward primer 5’-3’ | Reverse primer 5’-3’ |
| --- | --- | --- | --- |
| a (-7,7 kb) | CCCTTCTGCTGTGTGTGTGAGGACA | AAGGGCTTGAGGGAGTCTATT | GAAGGCTTTCCCTACTCCATAG |
| b (-5,5 kb) | TGCACCTAGGCCCACTAGCCACA | GGGCTGCATCAAATTTCTACTC | CCAAAGGGACAACCACTGTAG |
| c (-1,3 kb) | CCAGCTGTCACCCATGGCC | ACTCCTCACAGCCTCAGCAT | TCTTCTCTCCGCAGTGTCC |
| d (TSS) | TCCTGCTCCATCAGCCTCTTCCC | ACGGGATTCCAGGTAAGGAG | GTGTCTTGTCTTATAGGTCACAGCA |

**Supplementary File 2e. Single cell qPCR oligonucleotide sequences**

| Gene symbol | 5’-FAM-TAMRA-3’ probe | Forward primer 5’-3’ | Reverse primer 5’-3’ |
| --- | --- | --- | --- |
| *ASCL2* | CTTCGCACCGCCCTCCCGCTC | GCCTTACAGAATGTGATCGCG | CTTTCTCACGCCCTCCTTCC |
| *BACH2* | CCCTTGCTCCCATCCCTGCA | CAGGAATGAGGAGAATCTTCAA | CCTTGTGACAGGTCAGTGAGTG |
| *BCL6* | TGGCCTCGCCGGCTGACAG | AGCAAGGCATTGGTGAAGACA | ATGGCGGGTGAACTGGATAC |
| *BHLHE40* | CCTTGCTGTCCTCGCTCCGC | GACCTACCAGGGATGTACCCT | CGGTGCGGCAATTTGTAG |
| *EOMES* | TCCACCGCCACCAAACTGAGA | GTGCCCACGTCTACCTGTG | AGCTCAAGAAAGGAAACATGC |
| *FOXP3* | CAACATGCGACCCCCTTTCACC | GAGTTCCTCCACAACATGGACT | ATGGTTTCTGAAGAAGGCAAAC |
| *GATA3* | CAGCCAGGAGAGCAGGGA | CTCATTAAGCCCAAGCGAAG | ATTCCTCCTCCAGAGTGTGGT |
| *HOPX* | CCACGCTGTGCCTCATCGCGG | GTCGACAAGCACCCGGATT | GGCGCTGCTTAAACCATTTCT |
| *ID3* | CGCCTGCGGGAACTGGTACCC | TTGCTGGACGACATGAACCA | ACGCGCTGTAGGATTTCCAC |
| *IFI44* | AGTGCCCACCAAAGCCTGATGC | TCTGTTTTCCAAGGGCATGT | GCCATCTTTCCCGTCTCTAA |
| *IRF1* | AAGGATGCCTGTTTGTTCCGGAGCTG | AAGCATGGCTGGGACATCA | TTTGTATCGGCCTGTGTGAATG |
| *IRF3* | CGGCACCAACAGCCGCTTC | CCACTCCCTTCCCAAACCT | TCACCTCGAACTCCCACTCT |
| *IRF4* | CTTCGCTCCCAGCCCAGCAG | CCACCCCTACACCATGACA | GACGTAGTCCCTCCAGCTTC |
| *IRF7* | TGATGGTCACGTCCAGCGCC | CCAGGCAGAGCCGTACCT | GGTGTCCCACCACCTTCTG |
| *KLF6* | TGGCAACAGACCTGCCTAGAGCTG | CACGAGACCGGCTACTTCTC | CTCGCTCTGGAGGTAACGTT |
| *MYC* | CTTGTTCCTCCTCAGAGTCGCTGCTGGT | TCCCCTACCCTCTCAACGACAG | AGCCTGCCTCTTTTCCACAG |
| *NR4A1* | CCTGGAGCTCTTCATCCTCCGCC | ATCCCTGGCTTTGCTGAGC | CCTGGCTTAGACCTGTACGCC |
| *PRDM1* | ACAGAGCAGTCTAAAGCAACCGAGCAC | CGGAGAGCTGACAATGATGAATC | GGGACATTCTTTGGGCAGAGT |
| *PRF1* | CCATGTGGTACACACTCCCCCGC | TGGAGTGCGGCTTCTACAGTT | GCCCTCTTGAAGTCAGGGTG |
| *RORA* | ATGTGCCGTGCCTTTGACTCTCAGA | GGTTCTCTAGAGGTGGTGTTTATCA | GGCATACTTCCCATCAAAGTACAC |
| *RORC* | CTGCAGCAGCGGCAACAGCAG | CTGGGCATGTCCCGAGATG | GAGGGGTCTTGACCACTGG |
| *RUNX3* | CTCTTCCCTCGCCCACTGCG | GCCAGGTTCAACGACCTTC | ACAGTGATGGTCAGGGTGAA |
| *SOX4* | CGCCCGAGGTGAGCGAGATGA | TCGAGTTCCCGGACTACTGC | ACTCGAGCCAGTCTCCCGA |
| *STAT1* | CAAGGAGCGAGAGCGTGCCC | GCTCCCTCTCTGGAATGATG | ACTGAACCGCAGCAGGAA |
| *STAT3* | AAGGACATCAGCGGTAAGACCCAGA | AGGCGTCACTTTCACTTGG | GCTGCTTTGTGTATGGTTCC |
| *STAT4* | CAACAGAGCCACATTCTCCATCAGACC | CCCATCTCAACAATCCGAAG | AACACCGCATACACACTTGG |
| *STAT6* | CCACTTGCCAATGCCTTTC | CCACTTTCAGACAAATACT | CTTAAACTTGAGTTCTTCC |
| *TBX21* | TGAGGTGAACGACGGAGAGCCA | AACAATGTGACCCAGATGATTG | TGAACTGGGTTTCTTGGAAAGT |
| *ZBTB7B* | CATGCCCGCACTGCCCAGCC | ACCAGGAACGACAAGCTGAA | AGGTCGTAGCTGTGCAGGAA |
| *ZEB2* | ACAAGCCAGGGACAGATCAGCACCA | GCTCGAGCGGCATATGGT | ACTTGCGATTACCTGCTCCTT |
| *ZNF683* | TTCCAGCTCTTCCGAGGTGACCA | ATGGCCCTGGGAGGTACA | ACCATGTCTGGAAGTGGTCTG |

**Supplementary File 2f. References for the gene transfer plasmids used in transcription factor knockdown experiments**

| **GIPZ lentiviral ShRNA (Dharmacon)** | | |
| --- | --- | --- |
| **Name** | **Gene name** | **Catalog** |
| Non-silencing |  | RHS4346 |
| Empty vector |  | RHS4349 |
| Hopx | *HOPX* | V3LHS_316700 |
| Runx3 | *RUNX3* | V3LHS_364942 |
| T-bet | *TBX21* | V3LHS_382005 |
| Eomes | *EOMES* | V3LHS_387686 |
| ThPOK | *ZBTB7B* | V3LHS_638813 |
