# Supplementary material for: The transcription factors Runx3 and ThPOK cross-regulate acquisition of cytotoxic function by human Th1 lymphocytes
Source: eLife. 2018 Feb 28;7:e30496. doi: 10.7554/eLife.30496 (PMC5844691; doi:10.7554/eLife.30496)
Supplement: Supplementary file 3. — Gene Set Enrichment Analysis (GSEA) was used to analyze the enrichment of DNA methylation profiles in the GeneSets expressed by T cell subsets. Five Perl scripts were created to prepare the file required for GSEA. [file elife-30496-supp3.docx]

**Method to analyse the correlation between gene expression and DNA methylation**

Gene Set Enrichment Analysis (GSEA) was used to analyse the enrichment of DNA methylation profiles in the GeneSets expressed by T cell subsets. Five Perl scripts were created to prepare the file required for GSEA. The scripts were used as follows:

A. Preparation of the DNA methylation file.

1. Run perl_script_1.pl with whole DNA methylation dataset. This script will generate a file (File A) including the gene name, the gene name associated with the probe, the delta beta value, the location on gene, the p-value and the adjusted p-value.

2. Run perl_script_2.pl on File A in order to remove the duplicates. This generates File B.

3. Run perl_script_3.pl on File B to remove the lines where the gene name associated with the probe is unknown (NA) and to relabel TSS1500, TSS200, 5’UTR and 1stExon as PROM (promoter). This generates File C

4. Run perl_script_2.pl on File C in order to remove the duplicates. This generates File D.

5. Run perl_script_4.pl on File D to generate 3 different files (PROM, BODY and 3prime). The gene names associated with the probes and delta beta values of the PROM file are used to create a ranked list file (.rnk file) for GSEA analysis (see http://software.broadinstitute.org/cancer/software/gsea/wiki/index.php/Data_format).

B. Conversion of the expression GeneSets in probe signatures for the analysis of the enrichment of DNA methylation profiles.

1. Run perl_script_5.pl on expression GeneSets and PROM file to associate each gene of the GeneSets to all possible methylation probes of the gene promoters. This generates a GeneSet file (.txt file) that is used in the enrichment analysis.
2. Use the .txt file to create a .gmx or .gmt file for GSEA analysis (see http://software.broadinstitute.org/cancer/software/gsea/wiki/index.php/Data_format).

# perl_script_1

use strict;

my$tib;

open (A, $ARGV[0]);

my @tib = <A>;

close A;

print "A:".$#tib."\n";

open (I, "> File_A_".$ARGV[0]);

foreach (@tib) {

if ($_=~/^([^\t]+)\t([^\t]+)\t([^\t]+)\t([^\t]+)\t([^\t]+)\t([^\t]+)\t([^\t]+)\t([^\t]+)\t([^\t]+)\t([^\t]+)\t([^\t]+)\t([^\t]+)\t([^\t]+)\t([^\t]+)\t([^\t]+)\t([^\t]+)\t([^\t]+)\t([^\t]+)\t([^\t]+)\t([^\t]+)\t([^\t]+)\t([^\t]+)\t([^\t]+)\t([^\t]+)\t([^\t]+)\t([^\n]+)\n/) {

my$probe=$1;

my$delta=$12;

my$pval=$14;

my$adjpval=$15;

my$gene_name=$19;

my$position=$21;

my@genes=split(';', $gene_name);

my@positions=split(';', $position);

for(my $i=0; $i<=$#genes; $i++) {

print I $genes[$i]."\t".$genes[$i]."_".$probe."\t".$probe."\t".$delta."\t".$positions[$i]."\t".$pval."\t".$adjpval."\n";

}

}

}

# perl_script_2

use warnings;

open (A, $ARGV[0]);

while (<A>) {

chomp $_;

$lines[$#lines+1]=$_;

}

close A;

open (Z, "> File_B_".$ARGV[0]);

undef %saw;

foreach (@lines) {

if (!exists($saw{$_})) {

$saw{$_}=1;

} else {

$saw{$_}++;

}

}

while(($keys, $values) = each(%saw)) {

if ($values>=1) {

print Z $keys."\n";

}

else {

}

}

close Z;

# perl_script_3

use strict;

my$tib;

open (A, $ARGV[0]);

my @tib = <A>;

close A;

open (I, ">"."File_C_".$ARGV[0]);

my$n=0;

for ($n=0; $n<=$#tib; $n++) {

if ($tib[$n]=~/^NA\t/) {

}

else{

#$tib[$n]=~s/\:\:([^\n]*)//gio;

$tib[$n]=~s/TSS1500/PROM/gio;

$tib[$n]=~s/TSS200/PROM/gio;

$tib[$n]=~s/5\'UTR/PROM/gio;

$tib[$n]=~s/1stExon/PROM/gio;

print I $tib[$n];

}

}

close I;

# perl_script_4

use strict;

my$tib;

open (A, $ARGV[0]);

my @tib = <A>;

close A;

print "A:".$#tib."\n";

open (I, ">PROM_".$ARGV[0]);

open (J, ">BODY_".$ARGV[0]);

open (K, ">3PRIME_".$ARGV[0]);

print I $tib[0];

print J $tib[0];

print K $tib[0];

my $count=0;

my$n=0;

for ($n=0; $n<=$#tib; $n++) {

if ($tib[$n]=~/\tPROM\t/) {

print I $tib[$n];

}

elsif($tib[$n]=~/\tBody\t/) {

print J $tib[$n];

}

elsif($tib[$n]=~/\t3\'UTR\t/) {

print K $tib[$n];

}

}

close I;

close J;

close K;

# perl_script_5

use strict;

my$tib;

open (A, $ARGV[0]);

my @tib = <A>;

close A;

print "A:".$#tib."\n";

open (B, $ARGV[1]);

my @tob = <B>;

close B;

print "B:".$#tob."\n";

open (I, "> Met_GeneSet_".$ARGV[0]);

foreach (@tib) {

if ($_=~/^([^\n]+)\n/) {

my$gene=$1;

print "A_".$gene."_B"."\n";

for(my $i=0; $i<=$#tob; $i++) {

if ($tob[$i]=~/^$gene\t([^\t]+)\t/){

my$probe=$1;

print I $probe."\n";

}

}

}

}

close I;
